# Supplementary material for: Yeast eIF4A enhances recruitment of mRNAs regardless of their structural complexity
Source: eLife. 2017 Nov 30;6:e31476. doi: 10.7554/eLife.31476 (PMC5726853; doi:10.7554/eLife.31476)
Supplement: Supplementary file 1. [file elife-31476-supp1.docx]

**SUPPLEMENTARY METHODS**

RNAs used in the study.

RNA 1 was transcribed using the T7 RNA Polymerase and a DNA template while RNAs 2-10 were transcribed from a plasmid digested with a restriction nuclease at the 3'-end of the desired DNA template (i.e. run-off transcription) exactly as described previously (Acker et al., 2007).

| **RNA** | **DNA Template name** | **DNA Template Sequence (5'-3')**  complementary "clamp" DNA oligomer annealed to the region in bold | **Resulting T7 transcribed RNA sequence (5'-3') purified by denaturing acrylamide gel electrophoresis** |
| --- | --- | --- | --- |
| **1** | midAUG 23 | TCGACTTTGTTGTTGTTGTTGTTCCATTTGTTGTTGTTGTTGTTGTTG**CCTATAGTGAGTCGTATTACATATGCGTGTTACC** | GGCAACAACAACAACAACAACAA**AUG**GAACAACAACAACAACAAAGUCGA |

| **RNA** | **Plasmid name** | **Cloning Vector** | **Digested for T7 Polymerase run-off transcription** | **Resulting RNA sequence (5'-3') made by T7 RNA Polymerase run-off transcription and purified by denaturing acrylamide gel electrophoresis** |
| --- | --- | --- | --- | --- |
| **2** | SL25-0 | pBluescript II KS (+) | SalI-HF | GGAAAGAAUUCACUUAAGCAACAAa**aUg**GAAACAAACAAACAAACAAACAAACAAAACUAGUCAACAACAACAACAACAACAACAACAACAACAACAACAACAACAACAACAACAAcaacaacaacaaCAAcaacaacaacaacaacaacaacaacaaCAAcaacaacaacaacaacaacaaCAAcaacaacaacaacaacaaCAACAACAACAACAAcaacaacaacaacaacaacaaacaaacaaaG |
| **3** | SL150-0 | pBluescript II KS (+) | SalI-HF | GGAAAGAAUUCACAACAACAACAACAACAACAACAACAACAACAACAACAACAACAACAACAACAACAACUUAAGCAACAACAACAACAACAACAACAACAACAACAAUUGAAACAACAACAACAACAACAACAACAACAACAACAAAAA**AUG**GAAACAAACAAACAAACAAACAAACAAAACUAGUCAACAACAACAACAACAACAACAACAACAACAACAACAACAACAACAACAACAACAACAAAG |
| **4** | CAA617 | pUC57 | SspI-HF | GGAACAACAACAACAACAACAACAACAACAACAACAACAACAACAACAACAACAACAACAACAAAAA**AUG**AGAGAACAACAACAACAACAACAACAACAACAACAACAACAACAACAACAACAACAACAACAACAACAACAACAACAACAACAACAACAACAACAACAACAACAACAACAACAACAACAACAACAACAACAACAACAACAACAACAACAACAACAACAACAACAACAACAACAACAAAAU |
| **RNA** | **Plasmid name** | **Cloning Vector** | **Digested for T7 Polymerase run-off transcription** | **Resulting RNA sequence (5'-3') made by T7 RNA Polymerase run-off transcription and purified by denaturing acrylamide gel electrophoresis** |
| **5** | CAA663 | pUC57 | SspI-HF | GGAAAGAAUUGCCAUCUUGGCAAUUCAACAACAACAACAACAACAACAACAACAACAACAACAACAACAACAACAACAAAAA**AUG**AGAGAACAACAACAACAACAACAACAACAACAACAACAACAACAACAACAACAACAACAACAACAACAACAACAACAACAACAACAACAACAACAACAACAACAACAACAACAACAACAACAACAACAACAACAACAACAACAACAACAACAACAACAACAACAACAACAACAACAACAACAAAAU |
| **6** | CAA659 | pUC57 | SspI-HF | GGAAACAACAACAACAACAACAACAACAACAACAACAACAACAAGAAUUGCCAUCUUGGCAAUUCCAACAACAACAACAAAAA**AUG**AGAGAACAACAACAACAACAACAACAACAACAACAACAACAACAACAACAACAACAACAACAACAACAACAACAACAACAACAACAACAACAACAACAACAACAACAACAACAACAACAACAACAACAACAACAACAACAACAACAACAACAACAACAACAACAACAACAACAACAACAACAAAU |
| **7** | FJZ617 | pBluescript II KS (+) | SalI-HF | GGAAAGAAUUCAACAACAACAACAACAACAACAACAACAACAACAACAACAACAACAACAACAAAAA**AUG**AGAGCCAAGUGGAGAAAGAAGAGAACUAGAAGACUUAAGAGAAAGAGACGGAAGGUGAGAGCCAGAUCCAAAUAAGCGGAUUAUGAGUAAAUAACUCUAAUUUUGUUUUAAAUUCUUUCAAGAGUAUCGUAAUGUCAUUGAUGAAUUAACAUGUUAGUUUCUAUUCUACCUCAUAAUGGAUCUAAAUUGCAUACUAAUCUCACGGUGGGGUGUAAACCAUUGCCUACUAUUUAUAUAGUGCUUUAUAUAUGUCUCACAUAGUUUAAUCAAUUGUCCGUUUUUUUGG |
| **8** | FJZ663 | pBluescript II KS (+) | SalI-HF | GGAAAGAAUUGCCAUCUUGGCAAUUCAACAACAACAACAACAACAACAACAACAACAACAACAACAACAACAACAACAAAAA**AUG**AGAGCCAAGUGGAGAAAGAAGAGAACUAGAAGACUUAAGAGAAAGAGACGGAAGGUGAGAGCCAGAUCCAAAUAAGCGGAUUAUGAGUAAAUAACUCUAAUUUUGUUUUAAAUUCUUUCAAGAGUAUCGUAAUGUCAUUGAUGAAUUAACAUGUUAGUUUCUAUUCUACCUCAUAAUGGAUCUAAAUUGCAUACUAAUCUCACGGUGGGGUGUAAACCAUUGCCUACUAUUUAUAUAGUGCUUUAUAUAUGUCUCACAUAGUUUAAUCAAUUGUCCGUUUUUUUGG |
| **RNA** | **Plasmid name** | **Cloning Vector** | **Digested for T7 Polymerase run-off transcription** | **Resulting RNA sequence (5'-3') made by T7 RNA Polymerase run-off transcription and purified by denaturing acrylamide gel electrophoresis** |
| **9** | FJZ659 | pBluescript II KS (+) | SalI-HF | GGAAACAACAACAACAACAACAACAACAACAACAACAACAACAAGAAUUGCCAUCUUGGCAAUUCCAACAACAACAACAAAAA**AUG**AGAGCCAAGUGGAGAAAGAAGAGAACUAGAAGACUUAAGAGAAAGAGACGGAAGGUGAGAGCCAGAUCCAAAUAAGCGGAUUAUGAGUAAAUAACUCUAAUUUUGUUUUAAAUUCUUUCAAGAGUAUCGUAAUGUCAUUGAUGAAUUAACAUGUUAGUUUCUAUUCUACCUCAUAAUGGAUCUAAAUUGCAUACUAAUCUCACGGUGGGGUGUAAACCAUUGCCUACUAUUUAUAUAGUGCUUUAUAUAUGUCUCACAUAGUUUAAUCAAUUGUCCGUUUUUUUGG |
| **10** | pSW104 | pUC19 | BamHI | GGAGACCACAUCGAUUCAAUCGAA**AUG**AGAGCCAAGUGGAGAAAGAAGAGAACUAGAAGACUUAAGAGAAAGAGACGGAAGGUGAGAGCCAGAUCCAAAUAAGCGGAUUAUGAGUAAAUAACUCUAAUUUUGUUUUAAAUUCUUUCAAGAGUAUCGUAAUGUCAUUGAUGAAUUAACAUGUUAGUUUCUAUUCUACCUCAUAAUGGAUCUAAAUUGCAUACUAAUCUCACGGUGGGGUGUAAACCAUUGCCUACUAUUUAUAUAGUGCUUUAUAUAUGUCUCACAUAGUUUAAUCAAUUGUCCGUUUUUUUGG |

Note: pUC plasmids do not have a T7 promoter, therefore it was included in the plasmid insert during cloning, 5' to the desired RNA sequence.
